# Efficacy and safety of iota-carrageenan nasal spray versus placebo in early treatment of the common cold in adults: the ICICC trial

**DOI:** 10.1186/s12931-015-0281-8

**Published:** 2015-10-05

**Authors:** R. Eccles, B. Winther, S.L. Johnston, P. Robinson, M. Trampisch, S. Koelsch

**Affiliations:** Common Cold Centre, Cardiff School of Biosciences, Cardiff University, Cardiff, UK; Respiratory Disease Study Center, University of Virginia, Charlottesville, VA USA; Airway Disease Infection Section & MRC & Asthma UK Centre in Allergic Mechanisms of Asthma, National Heart and Lung Institute, Imperial College, London, UK; Boehringer Ingelheim Pharmaceuticals Inc., Therapeutic Area Virology, Ridgefield, CT USA; Boehringer Ingelheim Pharma GmbH & Co. KG, Biometrics & Data Management, Ingelheim/Rhein, 55216 Germany; Boehringer Ingelheim Pharma GmbH & Co. KG, CHC Development, Medicine & Regulatory Affairs, Ingelheim/Rhein, 55216 Germany

**Keywords:** Iota-carrageenan, Common cold, Human rhinovirus, Clinical trial

## Abstract

**Abstract:**

Iota-carrageenan (I-C) is active against respiratory viruses *in vitro* and was effective as nasal spray in three previous clinical trials. The current trial served to further investigate I-C in patients with early common cold symptoms.

**Methods:**

This randomized, placebo-controlled, double-blind phase IV trial was conducted in 200 adult patients with self-diagnosed colds of <48 h’ duration that were confirmed by baseline cold symptom scores. Patients were to self-administer 0.12 % I-C or placebo spray (NaCl 0.5 %) four times daily for four to ten days and record symptom information for ten days. Common respiratory viruses were quantified by RT-PCR during pretreatment and on Day 3 or 4. The primary endpoint was the mean total symptom score (TSS) of eight cold symptoms on Days 2–4 (TSS_2–4_).

**Results:**

Patients in both treatment groups had similar baseline TSSs (mean TSS: 6.75 for I-C and 6.79 for placebo). Viruses were detected in baseline samples from 53 of 98 I-C patients (54.1 %) and 54 of 97 placebo patients (55.7 %). Mean ± SE for TSS_2–4_ was 5.78 ± 0.25 for I-C patients and 6.39 ± 0.25 for placebo (*p* = 0.0895). Exploratory analyses after unblinding (TSS_2–4_ excluding a patient with aberrantly high symptom scores [TSS_2–4, ex 1pt_]; mean of TSS over Days 1–4 [TSS_1–4_]; change in TSS_1–4_ relative to baseline [TSS_1–4, rel_]) demonstrated treatment differences in favor of I-C (*p* = 0.0364, *p* = 0.0495 and *p* = 0.0421, respectively). For patients with quantifiable rhinovirus/enterovirus at baseline, there was a trend towards greater reduction of virus load at Day 3 or 4 (*p* = 0.0958; I-C: 90.2 % reduction in viral load; placebo: 72.0 %). Treatments were well tolerated with no differences in adverse event rates.

**Conclusions:**

The primary endpoint did not demonstrate a statistically significant difference between I-C and placebo but showed a trend towards I-C benefit. Exploratory analyses indicated significant reduction of cold symptoms in the I-C group relative to placebo during the first four days when symptoms were most severe, and also substantiated I-C’s activity against rhinovirus/enterovirus.

**Trial registration:**

NCT01944631 (clinicaltrials.gov)

## Background

The common cold is caused by a variety of respiratory viruses, such as human rhinoviruses (HRV), coronaviruses, human enteroviruses (HEV), respiratory syncytial virus (RSV), parainfluenza viruses, or influenza viruses [1]. Rhinoviruses are the most common cause of respiratory tract infections in individuals of all ages. In adults, rhinoviruses cause approximately 50 % of common colds and up to 90 % of colds during the autumn epidemic season. Common colds are frequent illnesses in both children and adults; on average, adults report 2.5 episodes per year [2]. It has been estimated that the total economic impact of non–influenza-related viral upper respiratory tract infections approaches $40 billion annually [2], and such infections can result in serious and even life-threatening sequelae in patients with underlying illnesses such as asthma, COPD or immune compromise. With the exception of influenza and RSV, there are no vaccinations or anti-viral medicinal products available for treatment of infection with the viruses that cause the common cold.

Iota-carrageenan (I-C)—a sulfated polysaccharide found in some species of red seaweed (*Chondrus crispus*) — has demonstrated antiviral activity against respiratory viruses in cell culture and in animal models [3, 4]. The I-C polymer seems to bind directly to viruses, preventing viral attachment to host cells. *In vitro* and *in vivo* studies have demonstrated the effectiveness of I-C against several viruses such as HRV [4] and influenza A [3]. *In vitro* tests have established that I-C does not penetrate freshly excised bovine nasal mucosa, and therefore is not absorbed systemically (data on file, Marinomed Biotechnology GmbH). Carrageenan is generally recognized as safe (GRAS) for use in food and topical applications. Because the primary site of infection and replication of most cold-causing viruses is the nasal mucosa, it was speculated that early and targeted treatment of the nasal mucosa with I-C may block viral entry at the level of the respiratory mucosa, and interfere locally with the propagation of viral replication.

Therefore, a nasal spray containing 0.5 % saline and 0.12 % iota-carrageenan (I-C nasal spray) has been developed and registered as a medical device. This product has recently been licensed to Boehringer Ingelheim, the sponsor of the current study. Between 2008 and 2011, three randomized clinical trials (two in adults and one in children) were conducted comparing I-C nasal spray with saline solution (placebo). In all 3 trials, there were indications of efficacy, including significantly reduced cold symptoms [5]; positive effects on symptoms in patients in whom less co-medication or no co-medication was used [6]; significantly reduced viral loads [5–7]; and faster reduction of common cold symptoms [6, 7]. Treatments were safe and well tolerated [5–7].

The ICICC trial (ICICC: Iota-Carrageenan In Common Cold) was designed as a controlled evaluation of the safety and efficacy of I-C in the treatment of patients with early common cold symptoms. The effects of treatment on cold symptoms and the duration of the cold and on viral load were assessed.

### Methods

#### Trial design and patient population

The ICICC trial was conducted at the Common Cold Center, Cardiff, Wales, UK. It was a randomized, placebo-controlled, double-blind, two-arm parallel group trial, with the aim of investigating the efficacy of I-C nasal spray in comparison with placebo nasal spray for the treatment of early common cold in adults (≥18 years). The trial was registered under the clinicaltrials.gov accession number NCT01944631.

Volunteer patients were recruited by poster advertisements around the Cardiff University campus and by e-mail to the students and staff of Cardiff University. Based on the results of a previous trial [5], it was calculated that a sample size of 93 in each group would have 90 % power to detect the expected difference in means between the TSS_2–4_ scores of the groups (see below for definition of TSS_2–4_); it was therefore planned to include a total of 200 patients eligible for randomization. To be eligible for participation, patients were to have had common cold symptoms for ≤48 h before trial entry, based on self-reporting during the screening interviews. Patients were to rate eight common cold symptoms (headache, muscle ache, chilliness, sore throat, blocked nose, runny nose, cough, and sneezing) on a 0 to 3 rating scale [8] (0 = no symptoms, 3 = maximum severity) and were to have a total symptom score (the sum of these eight common cold symptoms at baseline) of ≤9. Patients were also to have a score of ≥1 for at least one of the following symptoms: sore throat, runny nose, or blocked nose which reflects the standard cold study design according to Jackson.

Inclusion criteria constructed similarly to the previous 3 clinical trials conducted with I-C. Patients with high symptom scores (≥9) were excluded in order to recruit only patients in the early stages of a cold and exclude subjects with later infections. This strategy was designed to start treatment early to optimize the chance to improve the clinical course of a cold. This early intervention strategy may have been partially responsible for lowering the power of the study, since the proportion of patients in the trial who did not develop full-blown colds was relatively high (reflected by only about 24 % rhinovirus-positive patients; see Results and Discussion).

#### Trial treatment and outcome measures

Patients were instructed to self-administer 1 puff (0.14 mL) of trial medication to each nostril 4 times per day. Trial medication was either I-C nasal spray (1.20 g iota-carrageenan/L in 0.5 % NaCl) or placebo (0.5 % NaCl). Both patients and investigators were blinded to treatment allocation. Treatment was to be mandatory for 4 days, and depending on patient preference, could be continued for up to 6 additional days, resulting in a maximum treatment duration of 10 days.

Patients were to record their symptoms once per day in the evening of Days 1–10 in a symptom diary, using the 0–3 rating scale for each of the eight cold symptoms (see above). The primary endpoint of the trial was the mean total symptom score (TSS) of those documented eight single cold symptoms calculated as the averaged daily sum over Days 2–4 (TSS_2–4_). Secondary endpoints included the mean of the daily sum of three systemic common cold symptoms (headache, muscle ache, and chilliness) over Days 2–4 (SSS_2–4_); the mean of the daily sum of 5 single local common cold symptoms (sore throat, blocked nose, runny nose, cough, and sneezing) over Days 2–4 (LSS_2–4_); area under the curve (AUC) of daily symptom scores over the 10-day period (AUC-TSS_1–10_) calculated as the sum of the eight single cold symptoms over Days 1–10; duration of the cold (as determined by patients’ answer in the diary to the question “Do you still have a cold?” at the end of each treatment day); and the patients’ assessment of efficacy as evaluated at the end of trial visit, at which patients answered the question “How effective was the treatment in relieving your common cold symptoms?” using a 0–4 scale (0 = poor, 1 = fair, 2 = good, 3 = very good, or 4 = excellent). Further pre-specified endpoints included TSS at each study day (Days 1–10) and viral load (change from baseline) on Day 3 or 4 for various virus types. After trial data were unblinded, three exploratory analyses were performed: assessment of TSS_2–4_ excluding a patient with aberrantly high symptom scores (TSS_2–4, ex 1pt_); the mean of the TSS over Days 1–4 (TSS_1–4_); and change in TSS_1–4_ relative to baseline (TSS_1–4, rel_) to adjust for the potential impact of baseline TSS (TSS_0_) on TSS_1–4_ (TSS_0_ scores ranged from 3.00–9.00 in the I-C group and from 2.00–9.00 in the placebo group). To calculate TSS_1–4, rel_, TSS_0_ was subtracted from TSS_1–4_ and the result was divided by TSS_0_. In addition, TSS_2–4_ scores were calculated for the virus positive subset and the HRV/HEV positive subset (see Analysis sets below for definition). Safety was assessed on the basis of incidence of treatment-emergent adverse events (AEs) and overall tolerability was assessed by the patients themselves and recorded by the investigator at the final visit.

#### Viral assessment

Respiratory viruses and viral load were determined from nasopharyngeal lavage samples obtained at baseline and on either Day 3 or 4 [9]. Viral presence was to be determined for all patients using both qualitative and quantitative tests for respiratory viruses. Real-time polymerase chain reaction (RT-PCR) was used for qualitative determinations of the presence of influenza A and B, picornaviruses, HRV/HEV, human bocavirus, human metapneumovirus, coronaviruses 229E, HKU1, NL63, and OC43, adenovirus, human parechovirus, respiratory syncytial virus, and parainfluenza virus types 1–4. Quantitative viral loads for HRV and HEV were determined using the panenterhino/Ge/08 assay [10, 11]. Quantitative viral loads for influenza A and B and coronaviruses 229E, HKU1, NL63, and OC43 were assessed according to Garbino et al. [12, 13]. The panenterhino/Ge/08 assay provided quantifiable HRV and HEV viral load results; however, in some patients, virus was detectable but viral loads were below the level of quantification of the test. Such patients were considered to be virus-positive, but non-quantifiable (that is, a qualitative positive result). The total number of virus-positive patients was obtained by summing up the number of patients who had a positive result on any qualitative assay, or who had a detectable quantitative (quantifiable or non-quantifiable) result on the panenterhino/Ge/08 assay.

#### Analysis sets

Two main analysis sets (the treated set and the full analysis set [FAS]) were evaluated. The treated set included all patients who were documented to have taken at least one dose of trial medication, and was used for safety analyses. The FAS included patients documented to have taken at least one dose of trial medication, for whom a baseline TSS was recorded, and who supplied data for assessment of the primary endpoint. The FAS was used for analysis of the primary and secondary endpoints. Three analysis subsets within the FAS were also defined after unblinding, and included 1) the virus positive subset (those patients whose nasal lavage specimens yielded a least one positive qualitative or a detectable quantitative [quantifiable or non-quantifiable] result at baseline); 2) the HRV/HEV positive subset (those patients whose lavage yielded a least a detectable [quantifiable or non-quantifiable] result on the panenterhino/Ge/08 assay at baseline); and 3) the quantifiable HRV/HEV subset (those patients who had a quantifiable result on the panenterhino/Ge/08 assay at baseline). These subsets were used for analysis of additional exploratory endpoints.

#### Statistical methods

The primary endpoint TSS_2–4_ was analyzed using an analysis of covariance (ANCOVA) adjusting for continuous covariates ‘TSS_0_’ and ‘treatment’. Differences between I-C and placebo treatment groups were assessed based on the adjusted means and the corresponding two-sided 95 % confidence intervals (CIs). The secondary endpoints SSS_2-4_, LSS_2–4_, and AUC-TSS_1–10_ (adjusted for TSS_0_ and treatment) were analyzed similarly. The three exploratory endpoints TSS_2–4, ex 1pt_, TSS_1–4_, and TSS_1–4, rel_ were analyzed in the same way as the primary endpoint, that is, by adjusting for ‘TSS_0_’ and ‘treatment’. For all the above endpoints, missing single symptom scores at baseline and at any post-baseline measurement time were replaced by zero, if at least one of the eight symptom scores was available. Thereafter, missing daily TSS, LSS and SSS data were replaced the last observation carried forward procedure.

The duration of the cold was examined by recording the time to loss of cold, defined by the first “no” response to the question “Do you still have a cold?” in the patient’s daily symptom diary. The log rank test stratifying for TSS_0_ was used to compare treatment groups. The endpoint variable was censored at day 10 if a patient did not recover from his or her cold. To test whether patients’ assessment of efficacy differed between the I-C group and the placebo group, an ordinal logistic regression model adjusting for TSS_0_ was applied to the efficacy score (0–4) provided by patients. Missing entries were assigned the least favorable category.

The analysis of the endpoint variable TSS at each study day (Days 1–10) used a restricted maximum likelihood-based repeated measures approach, using all longitudinal TSS data from Days 1–10. The statistical model included the fixed categorical effects of treatment, day and treatment-by-day interaction and the continuous covariate of TSS_0_. An unstructured covariance structure was used to model within-patient errors. Missing single symptom scores at baseline and at any post-baseline measurement time were replaced by 0, if at least one of the 8 symptom scores was available. If a patient had a missing TSS at a specific day and all subsequent days until Day 10 and the cold ended on that day (that is, the question “Do you still have a cold” was answered by “no” at that day at latest), the missing TSS values for that and the subsequent days until Day 10 were replaced by 0. For all other cases no imputation of missing TSS values was performed.

The mean change in viral load from baseline was adjusted for the continuous covariates ‘baseline viral load’ and ‘treatment’ using an ANCOVA. Differences between I-C and placebo treatment were assessed based on the adjusted means and the corresponding 95 % CIs, which were calculated using a two-sided approach. For quantitative viral load analyses, missing values and values below the limit of quantification (LOQ) were replaced with the LOQ value.

## Results

**Fig. 1:**
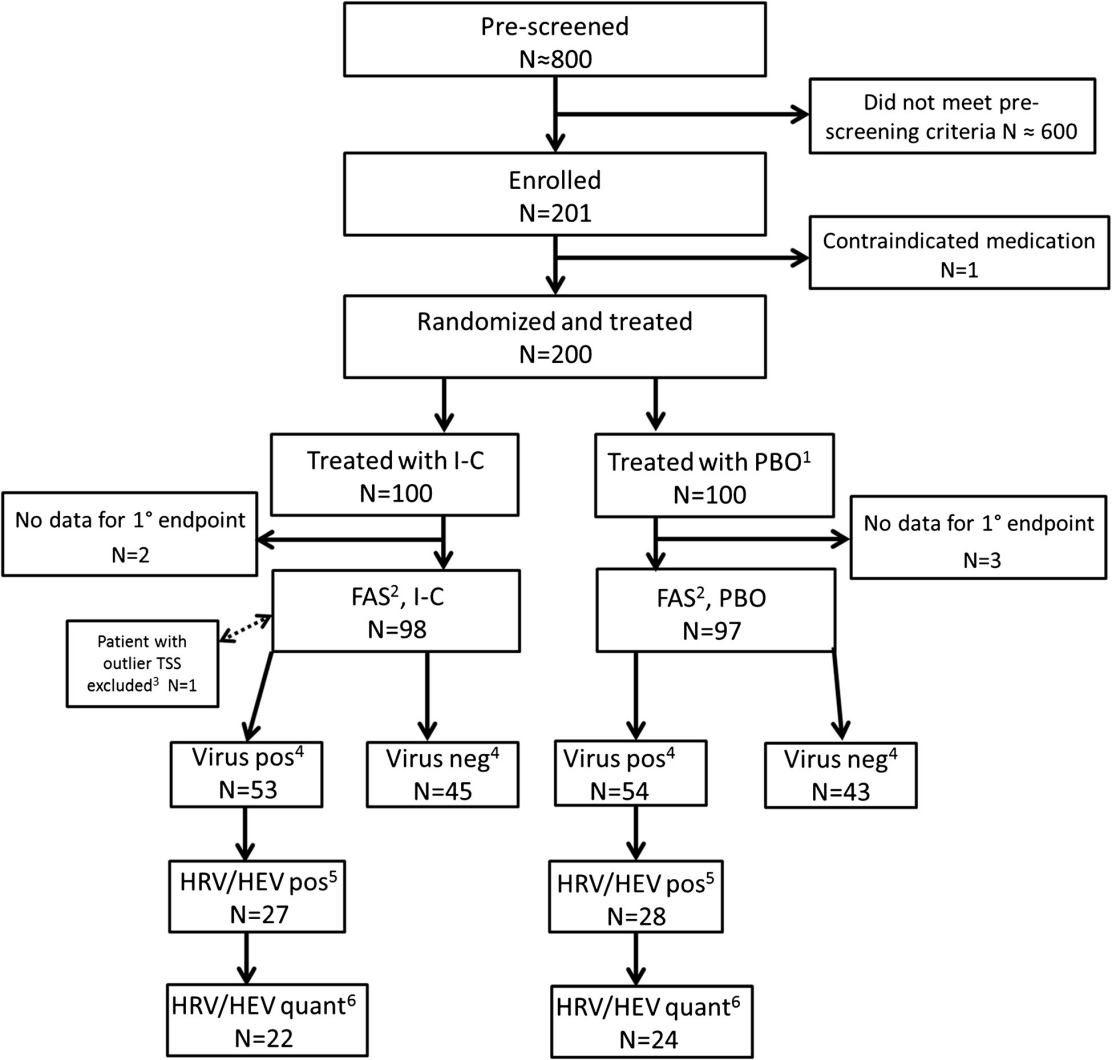
Disposition of trial patients. ^1^Placebo (PBO), ^2^Full analysis set, ^3^One patient with extreme outlier TSS baseline scores was excluded from some exploratory analyses, ^4^Virus positive patients were those with a positive qualitative test at baseline and/or a detectable quantitative (quantifiable or non-quantifiable) result on a quantitative test at baseline, ^5^Defined as a detectable quantitative (quantifiable or non-quantifiable) result on panentorhino/Ge/08 assay, ^6^Quantifiable result on panenterhino/Ge/08 assay

Approximately 800 patients were evaluated with respect to symptomatology and duration of symptoms (prescreening); of these, 201 were formally enrolled and screened. Of the enrolled patients, one was not randomized, and 200 were randomized and treated (I-C, *N* = 100; placebo, *N* = 100). Overall, 97.0 % of the 200 entered patients completed trial treatment, and 3.0 % prematurely discontinued treatment with trial medication. The proportion of patients discontinuing treatment was similar for the treatment groups (I-C: 2 of 100 patients, 2.0 %; placebo: 4 of 100 patients, 4.0 %). The most frequent reason for trial discontinuation was being lost to follow-up (I-C: 1 patient; placebo: 3 patients). Of the 200 treated patients, 98 in the I-C group and 97 in the placebo group provided data for the primary endpoint and were included in the FAS (Fig. 1).

Including the 200 patients who were treated, baseline demographic characteristics were comparable between the I-C and placebo groups (Table 1).

**Table 1:** Demographic characteristics – treated set

	I-C	Placebo	Total
Number of patients, *N* (%)	100 (100.0)	100 (100.0)	200 (100.0)
Sex, *N* (%)			
Male	40 (40.0)	38 (38.0)	78 (39.0)
Female	60 (60.0)	62 (62.0)	122 (61.0)
Race, N (%)			
Asian	8 (8.0)	2 (2.0)	10 (5.0)
Black or African American	2 (2.0)	1 (1.0)	3 (1.5)
Hawaiian or Pacific Islander	0	1 (1.0)	1 (0.5)
White	90 (90.0)	96 (96.0)	186 (93.0)
Age, mean (SD) [years]	20.01 (2.37)	19.93 (1.90)	19.97 (2.14)
BMI, mean (SD) [kg/m^2^]	23.98 (3.67)	23.66 (3.89)	23.82 (3.78)
Smoking history, N (%)			
Never smoked	80 (80.0)	83 (83.0)	163 (81.5)
Ex-smoker	7 (7.0)	4 (4.0)	11 (5.5)
Current smoker	13 (13.0)	13 (13.0)	26 (13.0)
Alcohol history, N (%)			
Does not drink	5 (5.0)	4 (4.0)	9 (4.5)
No significant drinking	95 (95.0)	96 (96.0)	191 (95.5)

*BMI* body mass index, *SD* standard deviation

### Primary endpoint

The primary endpoint of TSS_2–4_ was lower for the I-C group (5.78, 95 % CI: 5.28–6.28) than for the placebo group (6.39, 95 % CI: 5.89–6.89), but the difference was not statistically significant (−0.61, 95 % CI: −1.32–0.10; p-value 0.0895; Table 2).

**Table 2:** Total symptom scores over Days 2 to 4 (TSS_2-4_), ANCOVA-adjusted means, FAS

			I-C vs. placebo		
	N	Adjusted^a^ mean (SE)	Adjusted^a^ mean (SE) difference^b^	95 % CI	*p*-value*
TSS_0_^c^					
I-C	98	6.75 (0.17)			
Placebo	97	6.79 (0.18)			
TSS_2-4_					
I-C	98	5.78 (0.25)	−0.61 (0.36)	(-1.32, 0.10)	0.0895
Placebo	97	6.39 (0.25)			

*CI* confidence interval, *SE* standard error

*For ANCOVA-adjusted comparison of placebo with I-C

^a^The means were adjusted for TSS_0_

^b^A negative treatment difference favored I-C

^c^TSS_0_ values are unadjusted means

### Secondary and other endpoints

For the secondary endpoints SSS_2–4_, LSS_2–4_, and AUC-TSS_1–10_, there were no statistically significant differences between I-C and placebo (Table 3).

**Table 3:** Secondary symptom score endpoints SSS_2-4_, LSS_2-4_, and AUC-TSS_1-10_, ANCOVA-adjusted means, FAS

			I-C vs. placebo		
	N	Adjusted^a^ mean (SE)	Adjusted^a^ mean (SE) difference^b^	95 % CI	*p*-value*
SSS_2–4_					
I-C	98	1.12 (0.10)	−0.18 (0.15)	(-0.46, 0.11)	0.2310
Placebo	97	1.30 (0.10)			
LSS_2–4_					
I-C	98	4.66 (0.22)	−0.44 (0.30)	(-1.04, 0.16)	0.1465
Placebo	97	5.10 (0.22)			
AUC-TSS_1–10_					
I-C	98	41.94 (2.19)	0.73 (3.10)	(-5.39, 6.85)	0.8148
Placebo	97	41.21 (2.20)			

*CI* confidence interval, *SE* standard error

*For ANCOVA-adjusted comparison of placebo with I-C

^a^The means were adjusted for baseline SSS, LSS, or TSS

^b^A negative treatment difference favored I-C

Mean duration of time to ‘no cold’ was equivalent between the groups, at 7.5 days for I-C and 7.4 days for placebo. The patients’ overall assessment of efficacy was similar for I-C and placebo (OR = 1.11, 95 % CI: 0.67–1.84, *p* = 0.6954). For both I-C and placebo, the most frequent patient assessment was ‘good’ (I-C: 33.7 %; placebo: 36.1 %) and the second most frequent assessment was ‘fair’ (I-C: 28.6 %; placebo: 33.0 %).

**Fig. 2:**
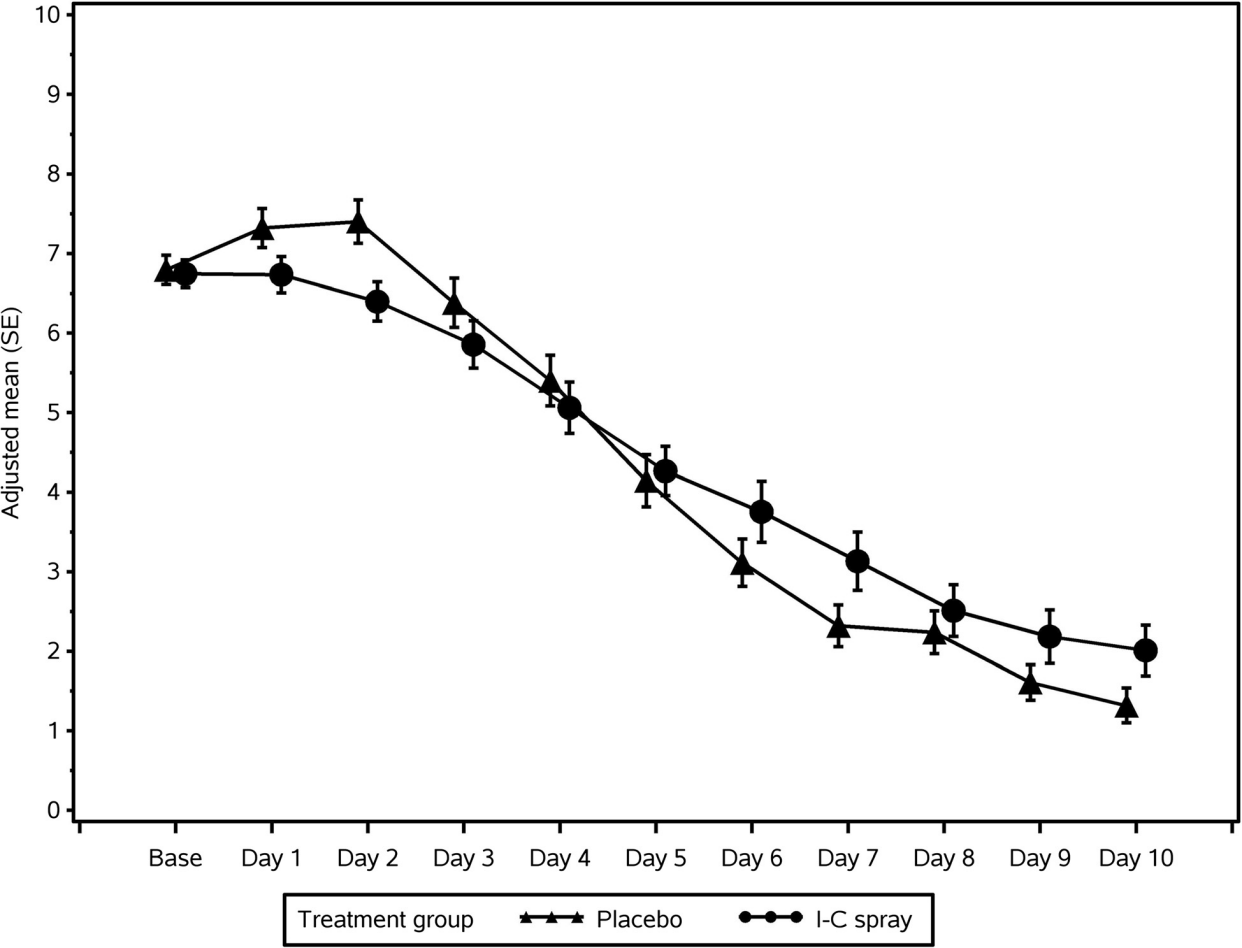
Adjusted mean TSS values with standard error for each day of the trial (data at baseline are displayed as unadjusted mean values)

For TSS at each study day (Days 1–10), the mean daily TSS values were highest for both groups during the four mandatory treatment days. The TSS peaked at Day 2 in the placebo group whereas the TSS in the I-C group decreased steadily without reaching a maximum on Day 2 (Fig. 2). TSS values were lower for the I-C group than for the placebo group on Days 1–4 (mandatory treatment days); the largest differences were observed on Days 1 and 2 of treatment. After Day 4, the TSS values were lower for the placebo group than for the I-C group.

### Exploratory analyses of efficacy

After unblinding, three previously unplanned analyses were performed to provide a better understanding of the primary endpoint. The data for a patient with a TSS_2-4_ of 16.7 (treated in the I-C group) was removed for an exploratory analysis, because his on-treatment symptom scores were well out of range when compared with the adjusted mean values of 6.39 for placebo and 5.77 for I-C patients (Table 2), suggesting that the patient may have misunderstood the process of symptom reporting. This aberrantly high symptom score was evident over the entire treatment period of 10 days. Furthermore, no respiratory viruses were detectable in his nasopharyngeal samples. Excluding the data for this patient resulted in an adjusted mean TSS_2–4_ that was statistically significantly lower for the I-C group (TSS_2–4, ex 1 pt_) compared with the placebo group (*p* = 0.0364; Table 4).

**Table 4:** Exploratory analyses of total symptom scores, ANCOVA-adjusted means

			I-C vs. placebo		
	N	Adjusted^a^ mean (SE)	Adjusted^a^ mean (SE) difference^b^	95 % CI	*p*-value*
TSS_2–4, ex 1 pt_					
I-C	97	5.67 (0.24)	−0.72 (0.34)	(−1.40, −0.05)	0.0364
Placebo	97	6.39 (0.24)			
TSS_1-4_					
I-C	98	6.02 (0.21)	−0.60 (0.30)	(−1.19, −0.00)	0.0495
Placebo	97	6.62 (0.21)			
TSS_1–4, rel_					
I-C	98	−0.08 (0.05)	−0.13 (0.06)	(−0.25, −0.01)	0.0421
Placebo	97	0.05 (0.05)			
Subset analyses					
TSS_2–4_, virus positive patients					
I-C	53	5.87 (0.34)	−0.80 (0.48)	(−1.75, 0.15)	0.0986
Placebo	54	6.67 (0.34)			
TSS_2–4_, HRV/HEV positive patients					
I-C	27	6.08 (0.46)	−0.36 (0.65)	(−1.66, 0.94)	0.5820
Placebo	28	6.44 (0.46)			

*CI* confidence interval

*For ANCOVA-adjusted comparison of placebo with I-C

^a^The means were adjusted for baseline TSS

^b^A negative treatment difference favours I-C

Because I-C binds to viruses and prevents their entry into cells [4, 5], an immediate effect on symptom scores could also reasonably be expected; in addition, the TSS for the placebo group was increasing from pre-treatment (baseline) to Day 2, indicating progression of the cold. Therefore, a sensitivity analysis was performed using the mean daily TSS over the mandatory treatment Days 1–4 (TSS_1-4_). For the adjusted mean TSS_1-4_, there was a statistically significant difference in favour of I-C (*p* = 0.0495; Table 4).

Finally, in order to adjust for the baseline severity of the cold, the TSS_1–4_ was analysed relative to baseline (TSS_1-4, rel_), yielding a statistically significant difference in favour of I-C (*p* = 0.0421; Table 4).

An exploratory analysis in the subset of 107 patients who tested positive for any virus at baseline yielded a trend in favor of I-C; average adjusted TSS_2–4_ scores were 0.80 points lower for the I-C group compared with placebo (*p* = 0.0986; Table 4). In the subset of 55 patients who were positive for rhinovirus/enterovirus at baseline, average adjusted TSS_2–4_ values were 0.36 points lower than placebo (*p* = 0.5820; Table 4).

### Virologic analyses

Including data from both qualitative and quantitative assays, the proportion of patients testing positive for virus at baseline was 54.1 % (53 of 98 patients) in the I-C group and 55.7 % (54 of 97 patients) in the placebo group (Table 5). In qualitative assays, the most frequent viruses identified were picornaviruses (I-C: 26.5 % of patients, placebo: 26.8 %) and coronaviruses (I-C: 22.4 %, placebo: 19.6 %; Table 5). Between baseline and Day 3 or 4, 7 of the 53 virus-positive patients in the I-C group converted to virus-negative (13.2 %), as did 8 of 54 patients in the placebo group (14.8 %). Conversely, 4 of the 44 patients in the I-C group (9.1 %) and 5 of the 43 patients in the placebo group (11.6 %) who were virus-negative at baseline converted to virus-positive by Day 3 or 4.

**Table 5:** Viruses identified at baseline in trial participants

	I-C	Placebo
Total number of patients tested (N)	98	97
Number of virus positive patients^a, b^ (N, %)	53 (54.1)	54 (55.7)
Influenza Type A	1 (1.0)	2 (2.1)
Picornavirus (including HRV and HEV)	30 (30.6)	28 (29.3)
Positive qualitative picornavirus assay	26 (26.5)	26 (26.8)
Positive qualitative enterovirus assay	2 (2.0)	0 (0.0)
Positive result^c^ on panenterhino/Ge/08 assay	27 (27.6)	28 (28.9)
Quantifiable result on panenterhino/Ge/08 assay	22 (22.4)	24 (24.7)
Human metapneumovirus	0 (0.0)	1 (1.0)
Coronavirus	23 (22.4)	19 (19.6)
Coronavirus 229E	1 (1.0)	2 (2.0)
Coronavirus HKU1	1 (1.0)	2 (2.1)
Coronavirus NL63	9 (9.2)	4 (4.1)
Coronavirus OC43	12 (12.2)	11 (11.3)
Adenovirus	0 (0.0)	2 (2.1)
Respiratory syncytial virus	0 (0.0)	1 (1.0)
Parainfluenza	5 (5.1)	5 (5.2)
Type 1	3 (3.1)	0 (0.0)
Type 2	1 (1.0)	1 (1.0)
Type 4	1 (1.0)	4 (4.1)

Categories are not exclusive; patients may appear in more than one category

^a^Eight patients were positive for 2 different viruses

^b^No patient tested positive for influenza B, human bocavirus, human paraecho virus, or parainfluenza 3

^c^Detectable (quantifiable or non-quantifiable) result at baseline

As a post-hoc analysis, the quantitative data for viral types identified in at least 2 patients in each treatment group at baseline were analysed (coronavirus NL63, coronavirus OC43, and quantified panenterhino/Ge/08 assay). For the viruses quantified with the panenterhino/Ge/08 assay, there was a more substantial reduction in virus load in the I-C group (90.2 % compared with 72.0 % for placebo), although this did not reach statistical significance (Table 6). For the coronaviruses, decreases in mean virus quantity from baseline to Visit 2 was similar between the placebo and I-C groups (reduction of 94 % or greater).

**Table 6:** Change in panenterhino/Ge/08 assay viral load in nasal secretions - patients with quantifiable baseline viral load, ANCOVA-adjusted means

			I-C vs. placebo		
	N	Adjusted^a^ mean (SE)	Adjusted^a^ mean (SE) difference^b^	95 % CI	*p*-value*
Baseline [log (copies/mL)]^c^					
I-C	22	5.840 (0.200)			
Placebo	24	6.139 (0.201)			
Change from baseline, [log (copies/mL)]					
I-C	22	−1.007 (0.191)	−0.453 (0.266)	−0.990, 0.084	0.0958
Placebo	24	−0.554 (0.183)			
Reduction from baseline (%)^c,d^					
I-C	22	90.2			
Placebo	24	72.0			

*CI* confidence interval, *SE* standard error

*For ANCOVA-adjusted comparison of placebo with I-C

^a^The means were adjusted for baseline viral load

^b^A negative treatment difference favored I-C

^c^Baseline values are unadjusted means

^d^Calculated according to Eccles *et al.* [5]

### Safety and tolerability

During this trial, there were no serious AEs or deaths. Twelve patients (6.0 %) reported an AE; the frequency was similar between treatment groups, at 5 of 100 patients (5.0 %) in the I-C group and 7 of 100 patients (7.0 %) in the placebo group. AEs of severe intensity were reported for 3 of 100 patients (3.0 %) in the I-C group (one patient with both headache and migraine, one patient with toothache, and one patient with malaise) and for 5 of 100 patients (5.0 %) in the placebo group (one patient each with headache, migraine, nausea, sore throat, and fatigue). One patient in the placebo group discontinued treatment due to an AE (moderate epistaxis). The patients’ assessment of tolerability was similar between treatment groups, with the majority rating the tolerability of the nasal spray as excellent, very good, or good (I-C: 87 patients, 88.8 %; placebo: 91 patients, 93.8 %). Only 1 patient in the I-C group (1.0 %) and 2 patients in the placebo group (2.0 %) assessed the tolerability of treatment as poor. Both treatments were therefore considered to be safe and well-tolerated.

## Discussion

This randomized, double-blind, and placebo-controlled ICICC trial was conducted to further evaluate the safety and efficacy of I-C in the treatment of patients with early common cold symptoms, and was conducted at one study site, primarily among university students who were in apparent good health except for early common cold symptoms. The analyses from the ICICC trial support the findings of three previous randomized clinical trials, which examined I-C nasal spray in adults [5, 7] and children [6] with acute common cold symptoms: in 35 adult patients, administration of I-C nasal spray significantly reduced cold symptoms and the viral concentration in nasal lavages [5]; in 203 adult patients, cold duration was significantly shorter in virus-positive patients treated with I-C, viral load was significantly reduced, and there was a significantly faster reduction of common cold symptoms after the first 3 days of treatment [7]; and in 153 children, although there was no significant difference between the I-C group and the placebo group for mean total symptom score, exploratory analyses indicated a positive effect on symptoms in patients in whom less co-medication or no co-medication was used [6]. In the ICICC trial, although the primary endpoint TSS_2–4_ did not demonstrate a statistically significant difference between I-C and placebo, there was a clear trend towards benefit of I-C treatment.

Consistent trends in favor of I-C for secondary endpoints and statistically significant outcomes for exploratory analyses were also observed for the ICICC trial. The finding that nominally greater treatment effects were seen in the ICICC trial among patients who were virus-positive, is supported by a pooled analysis by Koenighofer [14], which combined the Fazekas and Ludwig trials [6, 7]. The pooled analysis examined only those patients who were virus-positive, and showed that nominal response rates were higher in virus-positive patients than in all participants of the Ludwig and Fazekas trials.

There may be several reasons for the unexpectedly low power of the trial, which resulted in trends rather than statistically significant outcomes for the primary endpoint and HRV/HEV viral load reductions. First, the proportion of patients for whom a respiratory virus could be detected was smaller than anticipated; respiratory viruses (all groups/types analysed) were found at baseline in only 54.9 % of patients (54.1 % in the I-C group and 55.7 % in the placebo group). Efforts were made to recruit patients with the earliest onset of a common cold. Thus, self-reported cold symptoms were used for patient inclusion criteria, without the requirement for demonstration of virus positivity to enter treatment. Since the early symptoms of common colds may be rather non-specific for respiratory virus infection, these symptoms may present in other conditions such as vasomotor rhinitis, in which certain nonspecific stimuli including changes in environment (temperature, humidity, or barometric pressure), airborne irritants (odors or fumes), dietary factors (spicy food or alcohol) or emotional factors trigger nasal symptoms. Patients inadvertently included with non-infectious conditions would not be expected to respond clinically to antiviral therapy, and therefore are likely to cause an under-estimation of any true antiviral treatment effect. Consistent with this, the ICICC trial demonstrated that among those patients who had quantifiable virus at baseline, the I-C patients had a greater decrease in viral load than did placebo patients, suggesting an antiviral effect of I-C. In addition, TSS_2–4_ scores in the patients who were virus-positive at baseline showed strong trend in favor of I-C. However, since this was a subset of the total enrolled population, the virus-positive subset was underpowered for the TSS_2–4_ score endpoint. In particular, the percentage of patients with HRV/HEV identified at baseline was substantially smaller than anticipated: only 23.6 % had quantifiable virus (22.4 % in the I-C group and 24.7 % in the placebo group). Other studies using both molecular methods and viral culture have demonstrated that human rhinoviruses are the etiologic agent in 50–90 % of common colds [1, 15]. The current trial was also conducted primarily in the fall (October until February), which includes the period of greatest HRV infection incidence [1, 15]. Because virus shedding tends to be the greatest in the first several days of infection and generally continues for up to 2 weeks, it is unlikely that the low positive rate for HRV/HEV infection was due to missing the window of HRV shedding. More likely, the current study was conducted during a season of low HRV prevalence, resulting in fewer than expected infections among trial patients. In two previous clinical trials conducted with I-C [6, 7], the number of patients with confirmed HRV infection was higher than in the current trial, at 46 % [6] and 30 % [7] (although these values are as well lower than the percentages reported for other studies [1, 15]). Therefore, the smaller-than-expected number of patients infected with the HRV/HEV viruses likely limited the ICICC trial’s ability to differentiate between the I-C and placebo treatments.

A second issue that affected the analysis of the trial was the inclusion of one patient treated in the I-C group whose TSS scores were much higher than expected and were much different than those of other trial participants. This patient reported exceptionally high symptom scores over the entire treatment period of 10 days; his AUC-TSS_1–10_ was more than 3-fold higher than the overall population mean value. Interestingly, no virus was detected from this patient either at baseline or in a follow-up sample. While there is no ready explanation for these aberrant values, it is possible that this patient misinterpreted the symptom scoring methodology. When the primary endpoint TSS_2–4_ was re-evaluated excluding the data from this patient, the difference between the I-C and placebo patients became statistically significant.

Two other exploratory analyses further support the conclusion that I-C was efficacious. Because I-C may have had an early effect on symptoms, the assessment of on-treatment symptom scores was broadened to include Day 1. The comparison of TSS_1-4_ between I-C and placebo groups demonstrated a statistically significant effect of I-C, and a statistically significant change in TSS_1–4_ relative to baseline. Two additional exploratory analyses, although performed on very small samples, did not reach statistical significance, but also support the assertion of I-C efficacy: TSS_2–4_ scores in PCR-confirmed respiratory virus-positive patients were on average 0.8 points lower in the I-C group compared with placebo, and TSS_2–4_ scores in patients with PCR-confirmed rhinovirus infection were also on average 0.4 points lower in the I-C group.

The day-by-day TSS scores indicated an advantage for I-C on Days 1 and 2, which appeared to be lost by Days 4–5. It is not clear why the apparent TSS advantage disappeared on the later treatment days; however, the placebo was a saline nasal spray, which may have had some clinical benefit from saline irrigation of the nasal passages [16–18].

The I-C spray is registered and marketed as medical device in several European countries and is intended to be used in patients with early cold symptoms. Data from several experiments suggest that the mechanism of action may be prevention of binding to attachment sites and/or interference of viral entry. First, *in vitro* studies have demonstrated activity against a variety of viruses – various strains of HRV and influenza and herpesvirus, denguevirus, and papillomavirus [3, 4, 19–21]. This non-specificity suggests the absence of a specific direct-acting antiviral (DAA). Secondly, the observed antiviral effects occurred early in the infection cycle, most likely during the stages of attachment and/or entry, and the antiviral effect tended not to be I-C concentration-dependent. It is, therefore, unlikely that carrageenan’s effect is that of a DAA targeted to the metabolic or reproductive mechanisms. Since a variety of viruses cause the common cold and since the virus infection cycles typically involve binding and entry with nasal mucosal cells, the antiviral properties of iota carrageenan represents an attractive and promising option for treatment.

Published clinical evidence concerning the frequencies of detectable viruses varies but with modern techniques, viruses (particularly rhinoviruses) have been found in up to 90 % of patients suffering from cold symptoms. It can therefore be expected that in real-life conditions patients/consumers who empirically choose to use the I-C spray have a relatively high likelihood of being infected with rhinovirus or one of a variety of respiratory viruses that manifest with the common cold symptoms. The uncertainty of true infection in such a setting is counter-balanced by the very low intolerance and toxicity rates of the I-C spray and its relative inexpensiveness, thus giving favorable potential benefit-risk and benefit-cost ratios to the empiric use of the I-C spray.

In clinical trials of cough & cold, a substantial proportion of studies demonstrate trends but may not reach statistically significant efficacy outcomes. This observation may be due to the large variability in the disease severity between native infection and patients recruited in the early stages of a cold. When a controlled clinical trial tries to recruit patients at the earliest stages of a common cold - when symptoms are still emerging and are mild - patients may incorrectly believe they are coming down with a cold, prior to full blown cold symptoms. The relatively low frequency of rhinovirus-positive patients in the ICICC study demonstrates that there is often a trade-off when this standard design is used for cold studies. The peculiarities of the outcome of the ICICC study are also useful in that they may trigger a discussion among the scientific community about more suitable study designs to investigate common cold treatments.

Furthermore, the results of the ICICC study are in line with many other common cold studies which showed relatively small effects of treatment on symptom severity or duration. One example is the study by Barrett et al. on Echinacea which showed as well trends in the direction of benefit, amounting to an average half day reduction in the duration of a week-long cold, or an approximate 10 % reduction in overall severity. The authors concluded that “while these results do not allow us to reject the null hypothesis and confidently claim evidence-of-benefit, data are also insufficient to exclude the possibility of a clinically significant effect” [22].

Although not addressed in the ICICC trial, I-C’s antiviral properties lead to speculation that its use might decrease cold transmission to patients’ family members and other social contacts.

## Conclusions

Although the ICICC study failed to reach statistically significant outcomes for the primary and secondary endpoints, strong trends favouring treatment effect of I-C were observed. The primary and secondary endpoints consistently tended to be improved in the I-C spray group compared to the placebo group, with clinically relevant effect sizes, even though the population included patients with low baseline scores. Several rational and important exploratory analyses led to statistically significant and clinically relevant outcome differences. Unfortunately, the relatively low numbers of subjects with demonstrated viral infection limited study conclusions. Nonetheless, the outcome of the ICICC study is supported by previous evidence of I-C’s *in vitro* antiviral activity and symptom and viral reduction in prior cold studies. The ICICC trial’s outcome supports I-C spray as representing a potentially useful treatment option for the common cold. Trial results show it to be safe, well-tolerated, and minimally invasive, as well as suggesting efficacy.
